# PReS13-SPK-1283: Pathogenesis, diagnosis and management of neuropsychiatric SLE

**DOI:** 10.1186/1546-0096-11-S2-I4

**Published:** 2013-12-05

**Authors:** D Boumpas

**Affiliations:** 1 University of Athens, Athens, Greece

## 

Neuropsychiatric (NP) manifestations pose diagnostic and therapeutic challenges in systemic lupus erythematosus (SLE). Less than one-third of these events can be directly attributed to SLE; accordingly attribution to SLE remains a considerable challenge. Increased generalized SLE disease activity or damage, previous or concurrent major neuropsychiatric SLE (NPSLE) events, and persistently positive moderate-to-high antiphospholipid antibody titers are established risk factors. NPSLE patients have increased genetic burden and novel genomic approaches are expected to elucidate its pathogenesis. In animals with disturbed blood-brain barrier, autoantibodies against the NR2 subunits of the *N*-methyl-D-aspartate receptor and 16/6 idiotype antibodies, may cause diffuse NP manifestations through neuronal apoptosis or brain inflammation; data in humans are still circumstantial. In NPSLE, advanced neuroimaging uncovers structural and metabolic abnormalities in brain regions with normal appearance on conventional magnetic resonance imaging. The European League Against Rheumatism (EULAR) has published evidence and expert based opinion for the management of NPSLE(1).. According to them, diagnostic evaluation is guided by the presenting manifestation with MRI used to visualize brain or spinal pathologies. For neuropsychiatric events believed to reflect an immune or inflammatory process, or when these events occur in the context of active generalized disease, evidence (primarily from uncontrolled studies) supports the use of glucocorticoids alone or in combination with immunosuppressive therapy. In refractory cases, uncontrolled studies suggest a beneficial role of rituximab. Antiplatelet and/or anticoagulation therapy is recommended for NPSLE manifestations related to antiphospholipid antibodies, especially for thrombotic cerebrovascular disease. For the future, we anticipate that novel biomarkers and advanced neuroimaging tests will better define the underlying pathologic mechanisms of SLE-related neuropsychiatric disease, and help guide therapeutic decisions.

## Disclosure of interest

None declared.

